# Influence of the Use of an Ionic Liquid as Pre-Hydrodistillation Maceration Medium on the Composition and Yield of *Cannabis sativa* L. Essential Oil

**DOI:** 10.3390/molecules26185654

**Published:** 2021-09-17

**Authors:** Andrea Mezzetta, Roberta Ascrizzi, Marco Martinelli, Filomena Pelosi, Cinzia Chiappe, Lorenzo Guazzelli, Guido Flamini

**Affiliations:** 1 Department of Pharmacy, University of Pisa, Via Bonanno 6, 56126 Pisa, Italy; andrea.mezzetta@unipi.it (A.M.); Filly_Manu@hotmail.it (F.P.); cinzia.chiappe@unipi.it (C.C.); lorenzo.guazzelli@unipi.it (L.G.); 2 PlantLab, Institute of Life Sciences, Scuola Superiore Sant’Anna, Via Guidiccioni 8-10, 56010 San Giuliano Terme (PI), Italy; marco.martinelli@santannapisa.it

**Keywords:** dioecious cultivar Carmagnola, circular economy, distillation enhancement, 1,3-dimethyl-1H-imidazol-3-ium dimethylphosphate

## Abstract

*Cannabis sativa* L. is a multi-purpose crop, whose resilience, adaptability and soil-enriching properties make it a low-impact production. In the last years, the cultivation of the “industrial” hemp varieties (THC < 0.2%) has been promoted by many Countries, opening a whole new market of hemp-derived products, such as its essential oil (EO). Its distillation might represent an effective method to exploit a residue of the hemp fiber production (flowers), complying with the guidelines of the circular economy. In the present work, different concentrations of an ionic liquid (IL; 1,3-dimethyl-1H-imidazol-3-ium dimethylphosphate) have been studied as a pre-hydrodistillation maceration medium. The EO yields have been evaluated, and their compositions have been analyzed by GC-EIMS. The use of 100% and 90% IL concentrations gave a hydrodistillation yield increment of 250% and 200%, respectively. The 200% yield increase was maintained when the 100% IL was recycled after the hydrodistillation. The lower IL concentrations incremented the cannabinoid and oxygenated sesquiterpene contents, while the opposite was true for sesquiterpene hydrocarbons. The proposed IL-enhanced hydrodistillation medium applied to hemp, studied for the first time in the present work, might be used to both (i) noteworthy increment the hydrodistillation yield and (ii) modulate the obtained EO composition based on the desired final product.

## 1. Introduction

*Cannabis sativa* L. (Cannabaceae family) is an ancient species, reported as native to Central-Northeast Asia. Throughout the centuries, it has been used as a multi-purpose crop, undergoing numerous domestication and breeding processes aimed at selecting its most needed traits (e.g., seed yield, fiber development, etc.) based on the local production, which has led to the development of over 700 hemp cultivars [1].

Hemp is a resilient crop, characterized by low-management production requirements, great geographical adaptability, noteworthy resistance to parasites and pests, and soil-preserving properties, such as heavy metals phytoremediation ability [2,3,4,5]. All these traits make *C. sativa* an environment-friendly crop, as its production is low impact and does not deplete the soil. For this reason, many European countries, which had prohibited its cultivation in the fear of its use as an abuse drug, have recognized its unexploited potential as a multi-purpose crop and authorized the cultivation of the “industrial hemp” varieties, whose Δ^9^-tetrahydrocannabinol (THC) content is below 0.2% (threshold set by the EU legislation No. 2860/2000). Among these cultivars, one of the most used is Carmagnola, a dioecious hemp variety developed in Italy, and specifically selected for the fiber production.

The European Industrial Hemp Association (EIHA) 2018 report on European hemp cultivation and production evidenced as much as a 614% increment in the hemp-cultivated surface compared to 1993, with an increasing trend [6]. Even though the cultivation restrictions have relaxed, the commercialization of hemp-derived products is still very restrictively normed, up to completely lacking regarding some derivatives, one of which is *Cannabis sativa* L. essential oil (EO). As a value-added by-product, hemp EO could really represent the perfect final material to further exploit both flowers and leaves, which usually represent a waste material of the hemp fiber processing chain. This would not only reduce the cultivation waste material, but also its handling costs for the producers, complying with the requirements of the circular economy model. Many published studies reported its numerous, potential applications in different fields: its uses range from beverage flavoring agent [7], to insect pests repellent [8,9], acaricidal [10] and antimicrobial [11].

Even though easily obtainable and widely applicable, hemp EO has only one drawback: its distillation yield is low, mostly reported as lower than 0.5% *w/w* [2,12,13]. This depends on many factors, such as the genotype [12,14], the cultivation site [2,15], the agronomic characteristics [2,16,17,18], the developmental stage at harvesting [19], the storage conditions of the harvested material [12,14] and the used extraction procedure [20,21].

In the search for new media capable of ameliorating the efficiency of extraction processes, ionic liquids have attracted a great deal of interest in the last few years. Ionic liquids are organic salts which melt below 100 °C and are characterized by a negligible vapor pressure. This, combined with low flammability, made them an attractive alternative to traditional volatile organic solvents. Other intriguing properties of ionic liquids are their high thermal stability, the ability to dissolve a variety of inorganic [22] and organic [23] substrates, including biopolymers [24,25] and high thermal [26] and electrical conductivity [27]. Perhaps, the most outstanding feature of ILs is the possibility to tailor their properties on an application of interest by pairing the best matching ions, earning them the title of designer solvents. Hence, ionic liquids have found application in very disparate fields, such as organic reaction [28], electrochemistry [29], analytical chemistry [30], pharmaceutics [31] and chiral electroanalysis [32,33]. In the context of potential distillation enhancers, ionic liquids have been studied with promising results as adjuvants [34], aqueous biphasic systems [35,36] and hydrotropes [37] for the extraction of bioactive compounds such as astaxanthin [38], proanthocyanidins [39], syringic acid [40], artemisinin [37] and so forth.

Dealing with volatile compounds, published studies reported the successful use of ionic liquids as EO extraction-enhancers on different plant matrices. An hydrodistillation yield increase, with the same ionic liquid as maceration medium, was reported in the case of both a lignin-rich substrate (*Cinnamomum verum* J. Presl) [41] and a suberin-rich one (*Cuminum cyminum* L.) [42].

The present study aims at testing and developing an enhanced hydrodistillation method by using different concentrations of an ionic liquid (1,3-dimethyl-1H-imidazol-3-ium dimethylphosphate) as a pre-hydrodistillation maceration medium. For all the distillation conditions, both the EO yield and composition were evaluated. To the best of our knowledge, this is the first report on the use of an ionic liquid as a hemp EO distillation-enhancer.

## 2. Results

### 2.1. Essential Oil (EO) Compositions

The complete compositions of all the hydrodistilled essential oils (EOs) are reported in Table 1. Among all EOs, 72 individual compounds belonging to seven chemical classes were identified, accounting for 96.6% up to 99.1% of the total compositions.

Sesquiterpenes were detected as the most abundant compounds in all samples: Among them, hydrocarbons (SHs) prevailed in control (CTRL), macerated with only the ionic liquid both pure and after its recycling ([DMIM]DMP 100 and [DMIM]DMP 100 R1, respectively), and macerated with 90% ionic liquid and 10% distilled water ([DMIM]DMP 90); oxygenated sesquiterpenes (OSs), instead, were detected in higher relative abundances in the samples macerated with an 80:20 and 50:50 ionic liquid to water ratio ([DMIM]DMP 80 and [DMIM]DMP 50, respectively). Mirroring this chemical class behavior, β-caryophyllene, a SH, was the most abundant compound in the compositions of CTRL, [DMIM]DMP 100, [DMIM]DMP 100 R1 and [DMIM]DMP 90 samples, while its oxygenated counterpart (caryophyllene oxide) prevailed in the other ones. α-Humulene and its oxygenated counterpart, humulene oxide, exhibited the same distribution pattern in the EO compositions.

Monoterpene hydrocarbons (MHs) followed as the third most represented chemical class, as their relative concentration ranged from 5.5% ([DMIM]DMP 80) up to 21.0% ([DMIM]DMP 100 R1). Myrcene and α-pinene were detected as the most abundant MHs, followed by limonene, terpinolene and β-pinene. Oxygenated monoterpenes (OMs) exhibited statistically lower relative abundances in the two samples macerated with only the ionic liquid ([DMIM]DMP 100 and [DMIM]DMP 100 R1).

Cannabidiol and Δ^9^-tetrahydro-cannabinol were the only detected cannabinoids, whose relative abundance was statistically more significant in the two samples with the highest IL dilution ([DMIM]DMP 50 and [DMIM]DMP 80).

**Figure 1:**
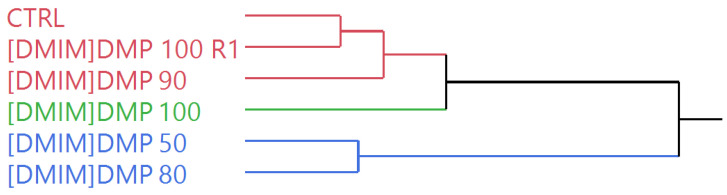
Dendrogram of the hierarchical cluster analysis (HCA) performed on the complete EO compositions of all samples.

The hierarchical cluster analysis (HCA), in which the closer the samples are reported in the dendrogram, the more similar they are on the considered basis of their complete EO compositions, distributed the samples in two macro-clusters, as shown in the dendrogram of Figure 1; (i) the first one, was composed of two sub-clusters (the red and the green one), (ii) the second macro-cluster was homogeneously constituted by the blue samples. The distribution appeared driven by the degree of dilution of the ionic liquid, as [DMIM]DMP 50 and [DMIM]DMP 80 were clustered by themselves. The samples with the highest ionic liquid concentrations ([DMIM]DMP 100, 100 R1 and 90) were, instead, clustered together with the control sample, thus evidencing a less significant compositional change compared to the blue samples.

**Figure 2:**
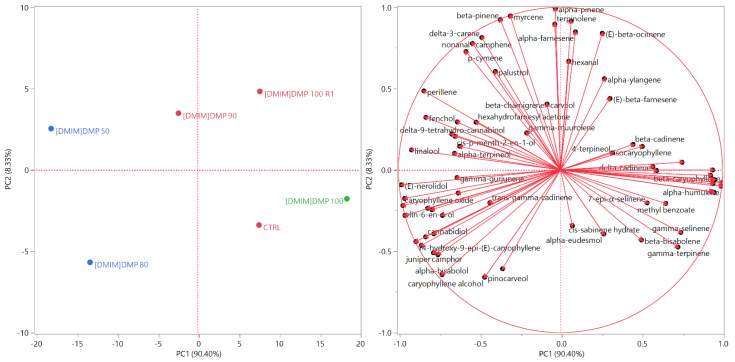
Score (**a**) and loading (**b**) plots of the principal component analysis (PCA) performed on the complete EO compositions of all samples.

This behavior was confirmed by the principal component analysis (PCA), as shown the score and loading plots of Figure 2, where the closer the samples are grouped, the more similar their compositions. The samples of the first macro-cluster identified by the HCA were plotted on the right quadrants (PC1 > 0) of the score plot, with the exception of [DMIM]DMP 90, which was positioned towards the right side of the upper left quadrant (PC1 < 0, PC2 > 0). These samples exhibited higher relative concentrations of β-caryophyllene and α-humulene, whose vectors point towards the right quadrants (PC1 > 0). The blue samples grouped by the HCA were, instead, both plotted in the left quadrants (PC1 < 0). As shown in the loadings plot (Figure 2), the vectors of both the identified cannabinoids point towards the left quadrants. Δ⁹-Tetrahydro-cannabinol lies on the upper left quadrant and, indeed, [DMIM]DMP 50 was the sample exhibiting the highest relative concentration for this compound. Cannabidiol, instead, lies on the lower left quadrant. Moreover, the vectors of the majority of the oxygenated terpenes (i.e., linalool, fenchol and carveol in the upper left quadrant; caryophyllene oxide and alcohol, juniper camphor and α-bisabolol in the lower left quadrant) were positioned in the left quadrants. The compositions of the blue samples were, indeed, richer in oxygenated terpenes compared to the samples of the first HCA macro-cluster.

### 2.2. Hydrodistillation Yields

**Figure 3:**
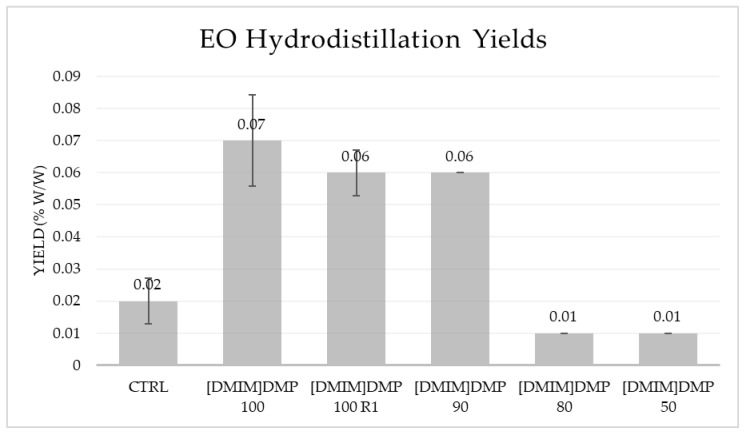
EO hydrodistillation yields (indicated as % *w*/*w*, vertical bars represent the standard deviations) of all samples.

The hydrodistillation yields for all samples are reported in Table 1 and Figure 3. The highest yield was found for samples [DMIM]DMP 100, 100 R1 and 90. These three samples yielded significantly more EO, showing a 250% increase in the case of [DMIM]DMP 100, and a 200% increment in the case of [DMIM]DMP 100 R1 and 90 compared to the control. The higher dilution of the ionic liquid of samples [DMIM]DMP 80 and 50, instead, caused a 50% decrease of the hydrodistillation yield compared to the CTRL sample.

## 3. Discussion

### 3.1. Essential Oil (EO) Compositions

The CTRL Carmagnola EO exhibited a composition differing from other samples reported in the literature: Even though these reports confirm the high relative abundance of compounds such as β-caryophyllene, α-humulene and caryophyllene oxide, monoterpene hydrocarbons (myrcene and α-pinene, in particular) were found in far more relevant relative quantities [12,13]. Another recent study, instead, reported an EO composition for a Carmagnola specimen mainly rich in cannabidiol [14]. This is not surprising, as Carmagnola has been reported as a quite heterogeneous hemp variety concerning its chemotype [43].

The influence of [DMIM]DMP used as maceration medium on the composition of EOs showed a concentration-dependent behavior. Cannabinoids and oxygenated sesquiterpenes were statistically more relevant in the samples macerated with higher dilutions of the ionic liquid ([DMIM]DMP 80 and 50). The same variation in the composition was reported for Carmagnola Selezionata (CS, a further selected Carmagnola, closely related to it) EOs obtained with a microwave-assisted distillation, compared to their Clevenger-hydrodistilled counterparts [20]. Fiorini et al. [20] hypothesized two mechanisms at the core of this cannabinoids and oxygenated sesquiterpenes increment: (i) a boosting-effect on the decarboxylation process which converts cannabidiolic acid into CBD, which ultimately increases the CBD content; (ii) an increment of the mechanical energy provided to the plant matrix, which leads to an increase in the distillation of the higher-boiling point sesquiterpenes over the lower-boiling monoterpenes. In the present case, the higher [DMIM]DMP dilutions (80 and 50), hence the higher water amount, might have loosened the interactions between ILs’ components (anion and cation) and some extracted compounds. This would cause a higher quantity of compounds being released during the hydrodistillation process which, ultimately, leads to (i) an incremented quantity of cannabidiolic acid available for the thermally-induced decarboxylation, and (ii) the augmented release of compounds (OSs) characterized by higher molecular weights. It is reasonable to assume that water, due to its amphiphilic nature, will have a greater effect on polar and large molecules. However, additional effects related to the change of viscosity of the medium due to the increased water amount cannot be excluded.

On the contrary, sesquiterpene hydrocarbons (SHs) were more quantitatively relevant in the composition of EOs obtained with the higher ionic liquid concentrations ([DMIM]DMP 100, 100 R1 and 90), which were, thus, more similar to the control sample.

### 3.2. Hydrodistillation Yields

The noteworthy hydrodistillation yield increases obtained with the lowest ionic liquid dilutions ([DMIM]DMP 100, 100 R1 and 90) seem to indicate the higher degree of disintegration of the plant matrix (in the case of *C. sativa* L., it is mainly composed of cellulose), which ultimately leads to a more significant release of EO from the plant cells. [DMIM]DMP is indeed one of the most effective ionic liquids for the dissolution of cellulose, while water acts as an antisolvent hampering the process [44,45]. The stability toward hydrolysis of [DMIM]DMP made its use particularly suitable for the hydrodistillation process [46]. The ^1^H NMR analysis of the recovered ionic liquid ensured the 1:1 ratio between anion and cation and, thus, its good quality.

A similar hydrodistillation yield increase was, indeed, also reported by Pistelli et al. [41] when using [DMIM]DMP diluted to 50% in water as maceration medium in the case *of Cinnamomum verum* J. Presl: In the latter case, however, the plant matrix was mostly rich in lignin. An increase in the EO hydrodistillation yield with a 50% dilution of [DMIM]DMP as maceration medium was also reported for *Cuminum cyminum* L. [42], a matrix mainly rich in suberin. This seems to indicate that, when solubilizing cellulose, lower water dilutions of [DMIM]DMP give better results compared to lignin and suberin, whose solubilization seems facilitated by lower concentrations of [DMIM]DMP as maceration medium. The increment of the hydrodistillation yield obtained with the use of the ionic liquid is particularly remarkable also when comparing it with those reported in the literature for specimens of this cultivar of different origins, as well as for other hemp varieties, for which it hardly reaches values of over 0.5% *w*/*w* [2,12,13].

To the best of our knowledge, this is the first report on the effect of this ionic liquid on both the composition and the hydrodistillation yield of hemp EO.

## 4. Materials and Methods

### 4.1. Plant Material and Growth Conditions

Seeds of *Cannabis sativa* var. “Carmagnola” (Canapuglia, Conversano (BA), Italy) were sown in 160 loam wells and incubated in a germination room at 24 °C for 2 days in the dark. Germination was achieved in controlled conditions in a greenhouse located in San Giuliano Terme (Pisa, Italy) at 22 °C, 16 h day/8 h night, 87.5 μEm^−2^ s^−1^. Homogeneous seedlings were transplanted into individual pots (10 cm diameter) containing 0.25 kg of a loam. Plants were cultivated in pots containing a biological substrate Brill Ortopack Bio (MT) (Agrochimica S.p.A., Bolzano, Italy) composed by blond peat (fraction 0–5), coconut fiber (light fraction) and black peat (fraction 0–6) with an apparent density of 270–320 g/L, an air volume of 20/25% and a water retention capacity of 5.8 g/g. The substrate was characterized as follows: pH 5.5–6.5; EC < 1 mS/cm; N 365 mg/L; P 125 mg/L; K 167.5 mg/L; Mg 12 mg/L; Fe 15 mg/L; S 38 mg/L. Plants were grown in the greenhouse from June to September at 30 °C day–20 °C night medium temperature, water irrigation was provided daily by aspersion applying a chemical fertilizer (NPK 8-12-10) every 20 days in the watering solution during the vegetative stage. Plants started flowering by the second week of August and fertirrigation was stopped. One week before harvest (September 15) watering was interrupted. Flowers and leaves were collected after 1 week from the treatment and drying was obtained in a naturally ventilated room at 20 °C for two weeks avoiding the exposition of the material to light. The drying was performed until the material reached constant weight; the measured residual moisture content was 9.60 ± 1.1 (% *w*/*w*).

### 4.2. NMR Analyses

NMR spectra were recorded with a Bruker Avance II (Bruker Italia S.r.l., Milano, Italy) operating at 400 (^1^H) and 100 MHz (^13^C) and using deuterated methanol and water as solvents. Chemical shifts (δ) are referenced to the residual solvent signal of D_2_O (^1^H 4.79 ppm) while coupling constants (*J*) are expressed in Hertz (Hz). The following abbreviations are used: s = singlet, m = multiplet, t = triplet, q = quartet.

### 4.3. 1,3-Dimethyl-1H-imidazol-3-ium Dimethylphosphate ([DMIM]DMP) Synthesis and Purification

1,3-Dimethyl-1H-imidazol-3-ium dimethylphosphate ([DMIM]DMP) was obtained following the general procedure previously reported for dimethylphosphate ionic liquids [47]. 1-Methylimidazole (1 equiv.) and trimethyl phosphate (1.05 equiv.) were mixed at room temperature under argon and then heated at 80 °C for 12 h without solvent. The obtained liquid was washed with anhydrous diethyl ether three times to remove the excess of reagent. Then, the recovered liquid was dried under vacuum for 2 h at 60 °C to afford a transparent liquid in an excellent yield (97%). The structure and the purity of [DMIM]DMP were checked by ^1^H-NMR analysis. ^1^H-NMR (D_2_O) δ 8.62 (s, 1H, *H*-2 IM), 7.39 (2× s, 2H, *H*-3 and *H*-4 IM), 3.85 (2× s, 6H, 2× C*H*_3_N), 3.53–3.50 (d, *J* = 12.0 Hz, 6H, 2× C*H*_3_OP).

### 4.4. Essential Oils (EOs) Maceration and Hydrodistillation

For each distillation, 50 g of ground dried plant material were subjected to a pre-hydrodistillation maceration with a maceration medium (the plant material:maceration medium ratio was 1:5) in a amber-glass bottle, and kept under mechanical agitation at 150 rpm for 24 h, at room temperature.

The maceration medium for each hydrodistillation was composed as follows:

1. Control (CTRL): 250 g of hydrodistilled water;
2. Pure 1,3-dimethyl-1H-imidazol-3-ium dimethylphosphate ([DMIM]DMP 100): 250 g of [DMIM]DMP;
3. Pure [DMIM]DMP recycled and purified after the previous hydrodistillation ([DMIM]DMP 100 R1): 250 of recycled and purified [DMIM]DMP;
4. 50% water, 50% [DMIM]DMP ([DMIM]DMP 50): 125 g of hydrodistilled water and 125 g of [DMIM]DMP;
5. 20% water, 80% [DMIM]DMP ([DMIM]DMP 80): 50 g hydrodistilled water and 200 g of [DMIM]DMP;
6. 10% water, 90% [DMIM]DMP ([DMIM]DMP 90): 25 g of hydrodistilled water and 225 g of [DMIM]DMP.

After 24 h, the macerated material was subjected to hydrodistillation in a standard Clevenger apparatus, equipped with a 2 L spherical flask, for 2 h. This distillation time was experimentally determined as the time interval after which no more essential oil (EO) could be obtained from the plant material. Each hydrodistilled EO was collected in an amber-glass vial and refrigerated until analysis.

### 4.5. Ionic Liquid Recycling

After the hydrodistillation nr. 2 (see Section 4.3), the residual material in the hydrodistillation flask, constituted by the biomass in [DMIM]DMP water solution, was roughly filtered. The obtained solution was then concentrated by removal of the water *in vacuo* at 60 °C for 2 h and then 80 mL of isopropanol were added to reduce viscosity. The suspension, composed of biomass particulate and ionic liquid alcoholic solution, was filtered under vacuum over celite on a Büchner funnel and then isopropanol was removed under vacuum at 40 °C for 3 h at reduced pressure to obtain the purified IL. The chemical structure of [DMIM]DMP was verified by ^1^H-NMR analysis.

### 4.6. Gas Chromatography-Electron Impact Mass Spectrometry (GC–EIMS) Analyses and Peak Identifications

Gas chromatography–electron impact mass spectrometry (GC–EIMS) analyses were performed with an Agilent 7890B gas chromatograph (Agilent Technologies Inc., Santa Clara, CA, USA) equipped with an Agilent HP-5MS (Agilent Technologies Inc., Santa Clara, CA, USA) capillary column (30 m × 0.25 mm; coating thickness 0.25 μm) and an Agilent 5977B single quadrupole mass detector (Agilent Technologies Inc., Santa Clara, CA, USA). Analytical conditions were as follows: injector and transfer line temperatures were set to 220 and 240 °C, respectively; the oven temperature was programmed to rise from 60 to 240 °C at 3 °C/min; helium was used as carrier gas, with a 1 mL/min flow; 1 μL of 0.5% HPLC grade *n*-hexane solution was injected; the split ratio was 1:25. The acquisition was performed in full scan, within a 30–300 *m*/*z* range, with a scan time of 1.0 s. The identification of the constituents was based on the comparison of their retention times with those of authentic samples, comparing their linear retention indices relative to the series of *n*-hydrocarbons. Computer matching was also used against commercial [48], our laboratory-developed mass spectra library, built up from pure substances and components of commercial essential oils of known composition, and MS literature data [49].

### 4.7. Statistical Analyses

The multivariate statistical analyses of the essential oil compositions and the ANOVA were performed with the JMP Pro 14.0.0 software package (SAS Institute, Cary, NC, USA). The hierarchical cluster analyses on all samples were carried out using Ward’s algorithm with Euclidean distances on normalized, unscaled data. The principal component analyses (PCA) were performed selecting the two highest principal components (PCs) obtained by the linear regressions operated on mean-centered, unscaled data [50]. For the principal component analysis, the data was a 72 × 6 covariance matrix (72 individual compounds × 6 samples = 432 data), with a total studied variance of 98.73% (of which 90.40% on PC1 and 8.33% on PC2). The observation of the groups of samples with the HCA and the PCA methods can be applied even without reference samples to be used as a training set to establish the model [51].

## 5. Conclusions

The low distillation yield of hemp EO is one drawback of its distillation as a value-added product in the exploitation of hemp flowers, a crop by-product which represents a waste material in the *C. sativa* fiber production process. As a solution to this limitation, the present work has demonstrated the efficacy of an ionic liquid, 1,3-dimethyl-1H-imidazol-3-ium dimethylphosphate ([DMIM]DMP), as a pre-distillation maceration method. High concentrations of this ionic liquid led to up to a 250% distillation yield increase in the case of the use in its pure form, and to a 200% distillation yield increment in the case of its 90% dilution in hydrodistilled water. The 200% increment was maintained even when the pure ionic liquid has been recycled after the first distillation round.

The influence of the ionic liquid on the EO composition was concentration-dependent: higher concentrations of [DMIM]DMP in the maceration phase caused an increment in the relative abundances of oxygenated sesquiterpenes and cannabinoids, while sesquiterpene hydrocarbons increased with higher dilutions of [DMIM]DMP.

To the best of our knowledge, this is the first report on the use of an ionic liquid as a distillation enhancer applied to hemp essential oil. Further studies are needed to assess if the same compositional and EO-yielding behavior is shown by other hemp varieties, or if this modulation is genotype-dependent. However, the proposed enhancement distillation method can be applied to both (i) increment the EO yield, and (ii) modulate the EO composition as needed, based on the expected final product.

## Figures and Tables

**Table 1:** Complete compositions of the hemp essential oil extracted from all the samples.

Compounds	l.r.i. ^1^	Relative Abundance (AVG ± SD)					
		CTRL	[DMIM]DMP 100	[DMIM]DMP 100 R1	[DMIM]DMP 90	[DMIM]DMP 80	[DMIM]DMP 50
hexanal	805	- ^2^	-	0.1 ± 0.03	0.2 ± 0.02	-	-
**α-pinene** ^3^	939	2.1 ± 0.71 ^BC^	2.8 ± 0.03 ^B^	5.4 ± 1.10 ^A^	5.4 ± 0.89 ^A^	0.9 ± 0.30 ^C^	4.9 ± 0.90 ^A^
camphene	954	-	-	0.1 ± 0.03	0.1 ± 0.02	-	0.2 ± 0.05
**β-pinene**	980	0.8 ± 0.28 ^B^	0.8 ± 0.00 ^B^	2.1 ± 0.39 ^A^	2.2 ± 0.34 ^A^	0.7 ± 0.20 ^B^	2.4 ± 0.38 ^A^
**myrcene**	991	2.1 ± 0.58 ^B^	2.2 ± 0.05 ^B^	7.6 ± 1.08 ^A^	6.7 ± 0.87 ^A^	1.9 ± 0.60 ^B^	7.5 ± 1.27 ^A^
δ-3-carene	1012	-	-	0.1 ± 0.02	0.2 ± 0.03	-	0.2 ± 0.03
*p*-cymene	1024	0.2 ± 0.04	0.2 ± 0.00	0.3 ± 0.04	0.4 ± 0.06	0.2 ± 0.06	0.5 ± 0.06
**limonene**	1031	2.2 ± 0.64 ^B^	2.2 ± 0.03^B^	2.9 ± 0.37 ^A,B^	2.7 ± 0.33 ^A,B^	1.2 ± 0.35 ^C^	3.0 ± 0.36 ^A^
1,8-cineole	1035	0.6 ± 0.18	0.8 ± 0.02	0.5 ± 0.07	0.5 ± 0.06	0.3 ± 0.07	0.3 ± 0.06
(*E*)-β-ocimene	1050	0.2 ± 0.04	0.2 ± 0.01	0.4 ± 0.04	0.2 ± 0.03	-	0.3 ± 0.02
γ-terpinene	1062	0.1 ± 0.01	0.1 ± 0.08	-	-	-	-
*cis*-sabinene hydrate	1070	0.1 ± 0.08	-	-	-	-	-
**terpinolene**	1089	1.2 ± 0.28 ^BC^	1.0 ± 0.03^C^	2.0 ± 0.18 ^A^	1.4 ± 0.14 ^B^	0.5 ± 0.11 ^D^	1.9 ± 0.13 ^A^
methyl benzoate	1091	-	0.2 ± 0.01	-	-	-	-
linalool	1100	0.3 ± 0.06	0.2 ± 0.03	0.3 ± 0.00	0.4 ± 0.06	0.4 ± 0.08	0.4 ± 0.03
perillene	1101	-	-	0.1 ± 0.07	0.1 ± 0.01	0.1 ± 0.07	0.2 ± 0.03
nonanal	1103	-	-	0.1 ± 0.01	0.1 ± 0.03	-	0.2 ± 0.02
fenchol	1115	0.3 ± 0.09	0.2 ± 0.01	0.3 ± 0.01	0.3 ± 0.04	0.3 ± 0.05	0.4 ± 0.02
*cis*-*p*-menth-2-en-1-ol	1123	0.2 ± 0.06	0.1 ± 0.11	0.2 ± 0.01	0.2 ± 0.04	0.2 ± 0.04	0.2 ± 0.00
*trans*-*p*-mentha-2,8-dien-1-ol	1125	-	-	-	0.2 ± 0.02	-	-
*cis*-*p*-mentha-2,8-dien-1-ol	1138	-	-	0.2 ± 0.00	0.4 ± 0.04	0.9 ± 0.18	0.4 ± 0.01
pinocarveol	1140	-	-	-	-	0.1 ± 0.07	-
*trans*-verbenol	1145	-	-	0.1 ± 0.08	-	0.1 ± 0.03	0.1 ± 0.01
(*E*)-tagetone	1146	0.1 ± 0.09	-	-	-	-	-
borneol	1167	0.1 ± 0.01	-	-	0.1 ± 0.10	0.1 ± 0.01	0.1 ± 0.09
4-terpineol	1178	0.4 ± 0.07	0.4 ± 0.05	0.2 ± 0.01	0.5 ± 0.03	0.2 ± 0.04	0.3 ± 0.00
*p*-cymen-8-ol	1185	0.2 ± 0.01	-	-	0.4 ± 0.01	0.3 ± 0.05	0.2 ± 0.02
α-terpineol	1190	0.4 ± 0.04	0.2 ± 0.02	0.3 ± 0.02	0.5 ± 0.03	0.4 ± 0.07	0.4 ± 0.00
carveol	1228	-	-	-	0.1 ± 0.01	-	-
α-ylangene	1372	-	-	0.1 ± 0.01	-	-	-
*iso*caryophyllene	1405	0.2 ± 0.01	0.3 ± 0.05	0.4 ± 0.06	0.3 ± 0.01	0.3 ± 0.00	0.1 ± 0.09
**β-caryophyllene**	1418	32.1 ± 0.68 ^B^	40.3 ± 1.42 ^A^	30.3 ± 1.00 ^B^	22.5 ± 0.54 ^C^	15.6 ± 1.27 ^D^	10.2 ± 0.06 ^E^
*trans*-α-bergamotene	1438	0.2 ± 0.06	0.5 ± 0.04	0.5 ± 0.03	0.3 ± 0.01	0.3 ± 0.00	-
α-guaiene	1440	0.3 ± 0.06	0.6 ± 0.02	0.4 ± 0.01	0.3 ± 0.01	0.2 ± 0.00	-
α-himachalene	1450	-	0.1 ± 0.13	-	0.1 ± 0.09	0.1 ± 0.00	-
**α-humulene**	1455	13.1 ± 0.13 ^B^	15.3 ± 0.47 ^A^	12.4 ± 0.43 ^C^	10.0 ± 0.22 ^D^	7.8 ± 0.45 ^E^	5.3 ± 0.04 ^F^
*(E)*-beta-farnesene	1459	-	0.2 ± 0.23	0.4 ± 0.06	0.3 ± 0.06	0.2 ± 0.02	-
***allo*aromadendrene**	1461	0.7 ± 0.04 ^C^	1.1 ± 0.05 ^A^	0.9 ± 0.04 ^B^	0.7 ± 0.01 ^C^	0.6 ± 0.02 ^D^	0.3 ± 0.00 ^E^
γ-gurjunene	1469	-	-	0.3 ± 0.01	0.2 ± 0.01	0.5 ± 0.02	0.2 ± 0.02
γ-muurolene	1476	-	-	0.1 ± 0.08	0.1 ± 0.00	0.1 ± 0.00	-
β-chamigrene	1477	-	-	-	0.6 ± 0.01	-	-
γ-selinene	1482	0.6 ± 0.06	0.9 ± 0.04	0.7 ± 0.08	-	0.5 ± 0.01	0.1 ± 0.19
**β-selinene**	1485	2.4 ± 0.13 ^B^	3.8 ± 0.01 ^A^	2.6 ± 0.17 ^B,C^	2.1 ± 0.02 ^B^	1.8 ± 0.02 ^B,C^	1.0 ± 0.01^C^
**valencene**	1492	1.9 ± 0.02 ^C^	3.4 ± 0.03 ^A^	2.3 ± 0.06 ^B^	1.7 ± 0.13 ^C^	1.4 ± 0.01^D^	0.7 ± 0.07 ^E^
α-bulnesene	1505	0.4 ± 0.04	0.8 ± 0.02	0.4 ± 0.02	0.3 ± 0.01	0.2 ± 0.04	0.1 ± 0.09
α-farnesene	1507	-	-	0.5 ± 0.04	0.3 ± 0.03	-	0.1 ± 0.08
β-bisabolene	1509	-	0.3 ± 0.04	-	-	0.1 ± 0.06	-
*trans*-γ-cadinene	1514	-	-	-	0.1 ± 0.07	0.1 ± 0.11	-
7-*epi*-α-selinene	1517	0.3 ± 0.01	0.5 ± 0.02	0.5 ± 0.13	0.4 ± 0.01	0.5 ± 0.04	0.1 ± 0.19
β-cadinene	1520	-	0.2 ± 0.00	-	0.2 ± 0.00	-	-
δ-cadinene	1523	-	0.2 ± 0.00	0.2 ± 0.21	-	0.1 ± 0.16	-
**selina-3,7(11)-diene**	1542	1.8 ± 0.35 ^D,E^	2.4 ± 0.04 ^D^	2.1 ± 0.07^C^	1.8 ± 0.04 ^B^	1.6 ± 0.11 ^A^	1.2 ± 0.09 ^E^
α-calacorene	1546	-	0.2 ± 0.04	-	0.1 ± 0.13	-	-
elemol	1549	0.4 ± 0.04	0.3 ± 0.01	0.3 ± 0.03	0.6 ± 0.01	0.8 ± 0.07	0.8 ± 0.04
(*E*)-nerolidol	1565	0.3 ± 0.06	-	0.3 ± 0.08	0.7 ± 0.01	1.1 ± 0.09	1.0 ± 0.11
palustrol	1568	0.5 ± 0.09	-	0.4 ± 0.13	0.5 ± 0.01	-	0.8 ± 0.06
caryophyllene alcohol	1569	-	-	-	-	0.9 ± 0.09	-
**caryophyllene oxide**	1582	12.8 ± 0.78 ^D^	7.3 ± 0.44 ^F^	10.2 ± 0.50 ^E^	15.9 ± 0.37 ^C^	23.6 ± 0.35 ^A^	22.0 ± 0.25 ^B^
viridiflorol	1590	-	-	-	0.2 ± 0.25	0.5 ± 0.13	0.2 ± 0.31
**humulene oxide**	1606	4.3 ± 0.16 ^D^	2.5 ± 0.09 ^F^	3.2 ± 0.16 ^E^	4.9 ± 0.04 ^C^	7.8 ± 0.10 ^A^	7.2 ± 0.15 ^B^
**selin-6-en-4-ol**	1618	1.7 ± 0.18 ^B^	1.0 ± 0.14 ^D^	0.9 ± 0.05 ^D^	1.3 ± 0.02 ^C^	1.8 ± 0.14 ^B^	2.4 ± 0.18 ^A^
1-*epi*-cubenol	1629	0.2 ± 0.22	-	0.2 ± 0.06	0.1 ± 0.11	0.7 ± 0.03	-
**caryophylla-4(14),8(15)-dien-5-ol**	1636	3.8 ± 0.37 ^B^	1.9 ± 0.37 ^C^	1.9 ± 0.21 ^D^	2.9 ± 0.07 ^C^	5.2 ± 0.25 ^A^	4.9 ± 0.35 ^A^
β-eudesmol	1649	-	-	-	-	-	0.2 ± 0.28
α-eudesmol	1653	0.4 ± 0.01	-	-	-	-	-
***neo*intermedeol**	1660	1.5 ± 0.09 ^A^	0.8 ± 0.21 ^B^	0.5 ± 0.05 ^B^	0.9 ± 0.02 ^B^	1.8 ± 0.16 ^A^	1.9 ± 0.47 ^A^
**14-hydroxy-9-*epi*-(*E*)-caryophyllene**	1665	1.7 ± 0.24 ^C^	0.7 ± 0.14 ^D^	0.8 ± 0.08 ^D^	1.4 ± 0.03 ^C^	3.0 ± 0.29 ^A^	2.6 ± 0.29 ^B^
α-bisabolol	1685	0.7 ± 0.08	0.5 ± 0.05	0.4 ± 0.01	0.5 ± 0.03	0.9 ± 0.13	0.8 ± 0.11
juniper camphor	1695	0.5 ± 0.07	0.3 ± 0.05	0.2 ± 0.01	0.4 ± 0.01	0.6 ± 0.04	0.6 ± 0.06
hexahydrofarnesyl acetone	1845	-	-	0.8 ± 0.12	0.8 ± 0.05	0.9 ± 0.14	0.4 ± 0.00
**cannabidiol**	2431	3.4 ± 0.49 ^B^	0.9 ± 0.13 ^C^	0.6 ± 0.04 ^C^	1.1 ± 0.01 ^C^	6.0 ± 0.90 ^A^	6.7 ± 0.39 ^A^
**Δ^9^-tetrahydro-cannabinol**	2468	0.1 ± 0.04 ^B^	- ^B^	- ^B^	- ^B^	0.1 ± 0.08 ^B^	1.3 ± 0.66 ^A^
**Monoterpene hydrocarbons**		8.8 ± 2.59 ^B^	9.5 ± 0.16 ^B^	21.0 ± 3.26 ^A^	19.3 ± 2.70 ^A^	5.5 ± 1.62 ^B^	20.8 ± 3.18 ^A^
**Oxygenated monoterpenes**		2.7 ± 0.67 ^A,B,C^	1.9 ± 0.20 ^C^	2.1 ± 0.06 ^B,C^	3.7 ± 0.45 ^A^	3.3 ± 0.76 ^A^	2.9 ± 0.09 ^A,B^
**Sesquiterpene hydrocarbons**		54.0 ± 0.11^B^	71.1 ± 1.55 ^A^	55.1 ± 2.11 ^B^	42.2 ± 1.16 ^C^	32.0 ± 1.23 ^D^	19.3 ± 0.81 ^E^
**Oxygenated sesquiterpenes**		28.7 ± 2.39 ^C^	15.4 ± 1.23 ^E^	19.3 ± 1.27 ^D^	30.1 ± 0.83 ^C^	48.8 ± 1.16 ^A^	45.3 ± 2.17 ^B^
**Apocarotenoids**		- ^D^	- ^D^	0.8 ± 0.12 ^B^	0.8 ± 0.05 ^A,B^	0.9 ± 0.14 ^A^	0.4 ± 0.00 ^C^
**Cannabinoids**		3.5 ± 0.54 ^C^	0.9 ± 0.13 ^D^	0.6 ± 0.04 ^D^	1.1 ± 0.01 ^D^	6.1 ± 0.98 ^B^	8.0 ± 0.27 ^A^
**Other non-terpene derivatives**		- ^C^	0.2 ± 0.01 ^B^	0.2 ± 0.02 ^B^	0.3 ± 0.05 ^A^	- ^C^	0.2 ± 0.02 ^B^
Total identified (%)		97.8 ± 0.44	99.1 ± 0.18	99.0 ± 0.11	97.5 ± 1.26	96.6 ± 1.33	96.9 ± 0.58
**Distillation yield** (% *w/w*)		0.03 ± 0.01 ^B^	0.06 ± 0.01 ^A^	0.07 ± 0.01 ^A^	0.06 ± 0.00 ^A^	0.01 ± 0.00 ^C^	0.01 ± 0.00 ^C^

^1^ Linear retention index on a HP-5MS capillary column; ^2^ Not detected; ^3^ For all elements reported in bold (compounds > 1% in at least one sample, all the detected chemical classes, and distillation yields), uppercase superscript letters (A–F) indicate statistically significant differences (established according to Tukey’s HSD *post-hoc* test, with *p* ≤ 0.05) among the samples.

## Data Availability

Not applicable.

## Sample Availability

Samples of the ionic liquid are available from the authors.
